# Epicardial adipose tissue defined by initial polytrauma CT of mechanically ventilated trauma patients: retrospective single-center cohort study to predict short-term outcomes

**DOI:** 10.1007/s10140-024-02242-0

**Published:** 2024-06-14

**Authors:** Hans-Jonas Meyer, Tihomir Dermendzhiev, Holger Kirsten, Michael Hetz, Christian Kleber, Timm Denecke, Michael Metze, Robert Werdehausen, Gunther Hempel, Manuel F. Struck

**Affiliations:** 1 Department of Diagnostic and Interventional Radiology, University Hospital Leipzig, Liebigstr.20, 04103 Leipzig, Germany; 2 Department of Orthopedics, Trauma and Plastic Surgery, University Hospital Leipzig, Liebigstr. 20, 04103 Leipzig, Germany; 3 Institute for Medical Informatics Statistics and Biometry, University of Leipzig, Härtelstr 16-18, 04107 Leipzig, Germany; 4 Department of Cardiology, Medical Department IV, University Hospital Leipzig, Liebigstr. 20, 04103 Leipzig, Germany; 5 Department of Anesthesiology and Intensive Care Medicine, University Hospital Leipzig, Liebigstr.20, 04103 Leipzig, Germany

**Keywords:** Trauma, Tracheal intubation, Epicardial adipose tissue, CT

## Abstract

**Purpose:**

Epicardial adipose tissue (EAT) detected by computed tomography (CT) is associated with morbidity and mortality in patients with COVID-19 and other critical care patient cohorts, whereas their prognostic relevance in trauma patients remains unclear. The present study explored associations with four potential short-term outcomes in trauma patients.

**Methods:**

All consecutive trauma patients requiring emergency tracheal intubation and mechanical ventilation before initial whole-body CT imaging at a level-1 trauma center over a 12-year period (2008–2019) were reanalyzed for this study. EAT was measured semiquantitatively in initial CT and analyzed regarding associations with 24-hour and 30-day mortality using Cox proportional hazard models. In survivors, associations of EAT with intensive care unit length of stay (ICU LOS) and mechanical ventilation duration were analyzed using linear regression analyses.

**Results:**

Four hundred fifty-five patients (74.7% male) with a median age of 49 years, and a median injury severity score (ISS) of 26 points were analyzed. In univariable analysis, EAT index was significantly associated with 24-hour and 30-day mortality (*p* = 0.007, and *p* = 0.013, respectively). After adjustment for significant predictors age, body mass index, and ISS, no significant associations were confirmed (*p* = 0.622, and *p* = 0.903, respectively). In a subanalysis of 353 survivors, EAT index was significantly associated with ICU LOS and mechanical ventilation duration in univariable analyses (*p* = 0.031, and *p* = 0.014, respectively), but not in multivariable analyses (*p* = 0.81 and *p* = 0.46, respectively).

**Conclusion:**

EAT index was associated with short-term outcomes in severely injured trauma patients, which not remained significant in multivariable analysis, suggesting that its prognostic capability is limited.

## Introduction

Whole-body computed tomography (CT) is the established imaging modality for emergency diagnostics and is performed particularly in severely injured trauma patients. It is capable of providing all necessary trauma findings covering the whole body within minutes [1].

In addition to the diagnostic capability of CT to detect acute life-threatening injuries, the assessment of the prognostic relevance of other CT-derived injury markers (e.g., body composition parameters and coronary artery calcification) has recently become increasingly popular [2–6].

Epicardial adipose tissue (EAT) was highlighted as an important CT-defined body composition parameter with a metabolic function, which is an anatomically and functionally distinct cardiac fat depot, situated entirely within the pericardial sac between the visceral pericardium and the epicardial surface of the heart. It is able to secrete inflammatory mediators in an autocrine or paracrine manner with prognostic relevance for cardiac disorders and metabolic syndrome [7].

Correlations between EAT and invasively measured hemodynamic parameters were demonstrated in patients with congestive heart failure suggesting that EAT was associated with right ventricular end-diastolic pressure [8]. Beyond that, the importance of EAT assessment was shown in acute diseases, comprising acute pulmonary embolism and COVID-19, demonstrating its relevance in critical ill patients [9, 10]. Data on the physiological implications of EAT and its prognostic relevance in severely injured trauma patients are not available.

The aim of the present study was to analyze the prognostic capability of EAT measurements derived from initial trauma CT in a cohort of trauma patients who underwent emergency tracheal intubation and mechanical ventilation. We hypothesized that EAT, as a representative factor of the general cardiovascular condition, would be prognostically relevant in severely injured patients.

## Materials and methods

### Patient acquisition

**Fig. 1:**
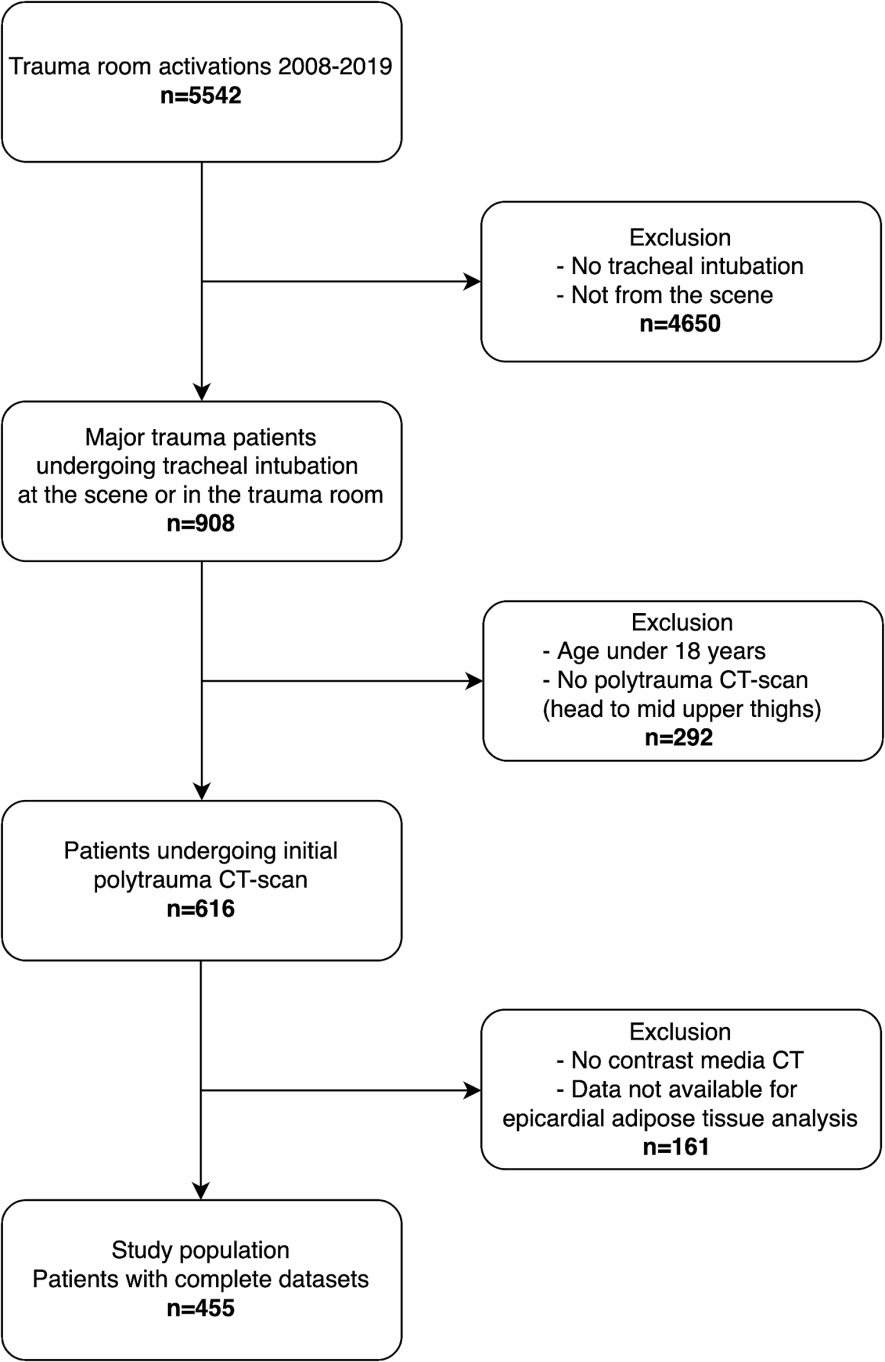
Study flow chart of the present study. Overall, 455 patients were included into the present analysis

After approval by the ethics committee at the Medical Faculty, Leipzig University, Leipzig, Germany (IRB00001750, project ID 441/15ek, September 14, 2020), consecutive trauma patients of the University Hospital Leipzig between January 2008 and December 2019 were retrospectively analyzed. Informed consent was waived by the ethics committee since only anonymous data were analyzed and published and all data was obtained according to the human rights declaration of Helsinki. The inclusion criteria were direct admission from the scene to the emergency department (ED), presence of severe injuries requiring emergency tracheal intubation, performance of initial emergency CT diagnostics and admission to the intensive care unit (ICU). The exclusion criteria were patients younger than 18 years, incomplete or missing data, and CT imaging without contrast media use. Full compliance with the Strengthening the Reporting of Observational Studies in Epidemiology (STROBE) guidelines of cohort studies was provided. Figure 1 shows the study flow chart.

### Investigated parameters

Demographic parameters included sex, age, and body mass index (BMI). Injury severity was classified using the injury severity score (ISS). ICU length of stay (ICU LOS) in days, mechanical ventilation duration in days, and all-cause 24-hour and 30-day mortality were assessed. All investigated parameters were obtained from paper-based and electronic patient charts and transformed into table format for further processing after anonymization of personal data.

### Imaging technique

Contrast-enhanced CT was performed in a clinical setting using a 128-slice CT scanner (Ingenuity 128, Philips Health care, Eindhoven, The Netherlands). Iodine-based contrast medium (90 mL Imeron 400 MCT, Bracco Imaging Germany GmbH, Konstanz, Germany) was administered intravenously at a rate of 2–4.0 mL/s. Automatic bolus tracking was performed in the descending aorta with a trigger of 100 Hounsfield units. CT images were obtained in the late arterial phase in every case. Typical imaging parameters were as follows: 100 kVp; 125 mAs; slice thickness, 1 mm; and pitch, 0.9. The CT covered the head to the upper thighs.

### Epicardial adipose tissue quantification

**Fig. 2:**
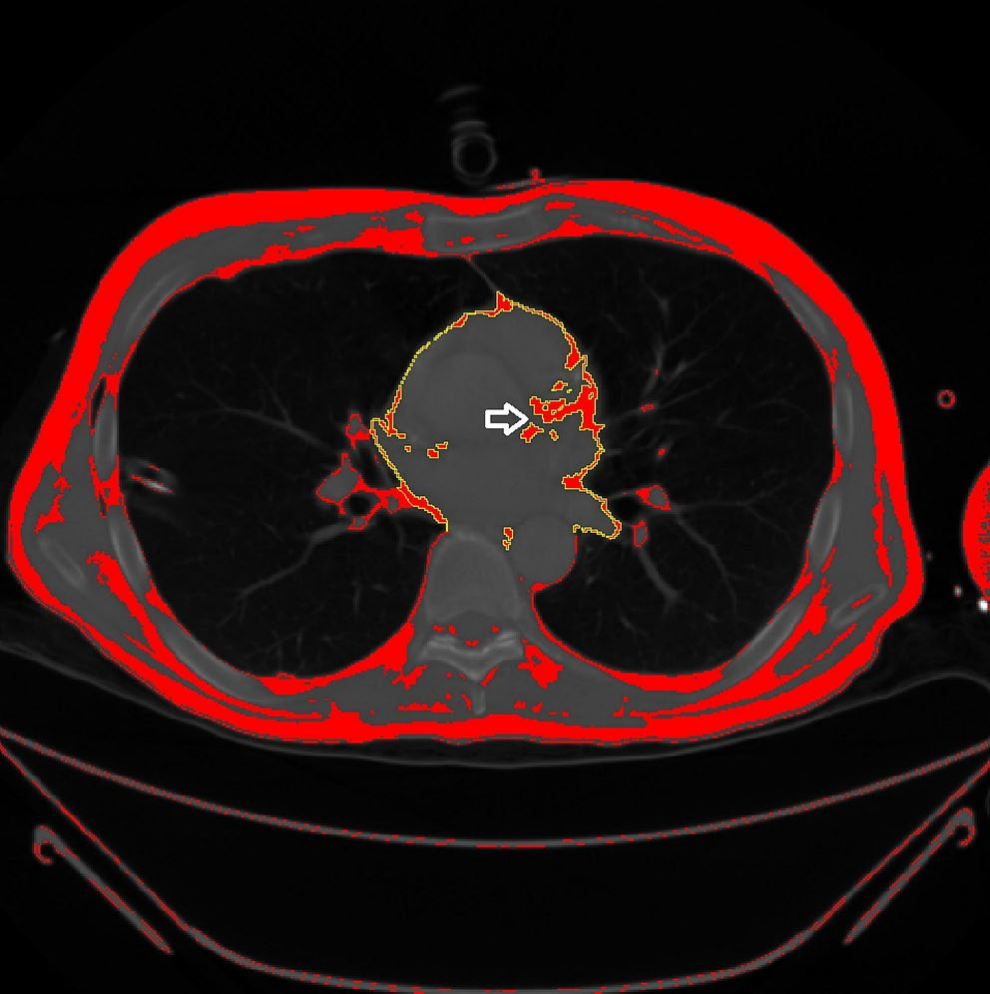
Representative 49-years old male patient of the patient cohort with an ISS score of 29. Axial CT slice with the measured EAT highlighted demonstrated in yellow. The arrow indicates the origin of the left main coronary artery

A trained radiologist, blinded to the patient outcomes, measured the EAT area semiautomatically with the freely available ImageJ software 1.48v (National Institutes of Health, Bethesda, Maryland, USA). The EAT area at the origin of the left main coronary artery was set at as the representative CT slice. This method was closely associated with the total volume of the entire EAT, as shown previously [9]. The EAT is localized by manually tracing the pericardium and extracting the area with CT values between – 230 HU and – 30 HU. The resulting area with cm² were adjusted for the body height squared, as performed previously [9]. This was performed to reduce possible bias induced by different body height. Figure 2 displays a representative measurement of one patient.

### Statistical analysis

Data analysis included absolute numbers and proportions and medians and interquartile ranges (IQR, quartile 1 and quartile 3). After testing for normality distribution with the Kolmogorov–Smirnov test, group differences were calculated with the Mann‒Whitney U test, Student’s t test, and Chi-square test when appropriate. Spearman’s correlation analysis was used to elucidate associations between EAT and clinical parameters. To identify independent predictors of 24-hour and 30-day mortality, the Cox proportional hazard model was applied in which statistically significant predictors of univariable analyses were included in the multivariable model. In survivors, associations with ICU LOS and mechanical ventilation duration were analyzed using multivariable linear regression analyses, which included statistically significant predictors of univariable analyses. Here, outcome variables were transformed using the natural logarithm as their original distribution was considerably right-skewed. This transformation leads to less biased and more efficient estimates in linear regression analysis. No significant collinearity was observed in multivariable models (all variance inflation factors VIF < 1.5). Hazard ratios, beta weights and 95% confidence intervals are provided. In all instances, p values < 0.05 were considered statistically significant. The statistical analysis was performed using R 4.2.2 (R Foundation for Statistical Computing, Vienna, Austria), DATAtab (DATAtab e.U., Graz, Austria) and GraphPad Prism version 10.0.2 for MacOS (GraphPad Software, Boston, Massachusetts, USA).

## Results

Overall, 455 patients (340 male patients, 74.4%) with a median (IQR) age of 49 (31–64) years met the inclusion criteria (Fig. 1; Table 1). Traffic accidents were the main cause of injury (59%), whereas 31% of the patients had falls from height, 7% had other blunt injuries, and 3% had penetrating injuries. The median ISS was 26 (20–41) points, the median ICU LOS was eight (3–22) days, and the median mechanical ventilation duration was three (0.5–14) days. The all-cause 24-hour mortality was 7.7% (35 patients) and 30-day mortality was 22.4% (102 patients) (Table 1).

**Table 1:** Baseline characteristics of the study cohort and comparison of survivors and non-survivors at 30 days

Parameter	All patients (*n* = 455)	Survivors (*n* = 353)	Non-survivors (*n* = 102)	*p* value
Male, n (%)	340 (74.7)	265 (75.1)	75 (73.5)	0.752
Age, years; median (IQR)	49 (31–64)	47 (31–61)	59.5 (35.5–74.8)	**< 0.001**
BMI, kg/m^2^; median (IQR)	25 (23–28)	25 (23–28)	26 (24–28)	**0.049**
ISS; median (IQR)	26 (20–41)	25 (18–34)	44 (34–57)	**< 0.001**
EAT, cm^2^; median (IQR)	7.7 (3.5–14.2)	7.3 (3.5–13.8)	9.4 (4.7–15.7)	**0.03**
EAT index, cm^2^/m^2^; median (IQR)	2.4 (1.2–4.5)	2.2 (1.1–4.2)	3.1 (1.5–5.2)	**0.016**
ICU LOS, days; median (IQR)	8 (3–22)	12 (4–27)	3 (1–7)	**< 0.001**
Mechanical ventilation, days; median (IQR)	3 (0.5–14)	4 (0.5–16)	3 (1–7)	**0.017**
24-hour mortality; n (%)	35 (7.7)		35 (34.3)	
30-day mortality; n (%)	102 (22.4)		102 (100)	

IQR, interquartile range; BMI, body mass index; ISS, injury severity score; EAT, epicardial adipose tissue; ICU LOS, intensive care unit length of stay. Bold numbers indicate statistical significance (*p* < 0.05).

### Epicardial adipose tissue

**Fig. 3:**
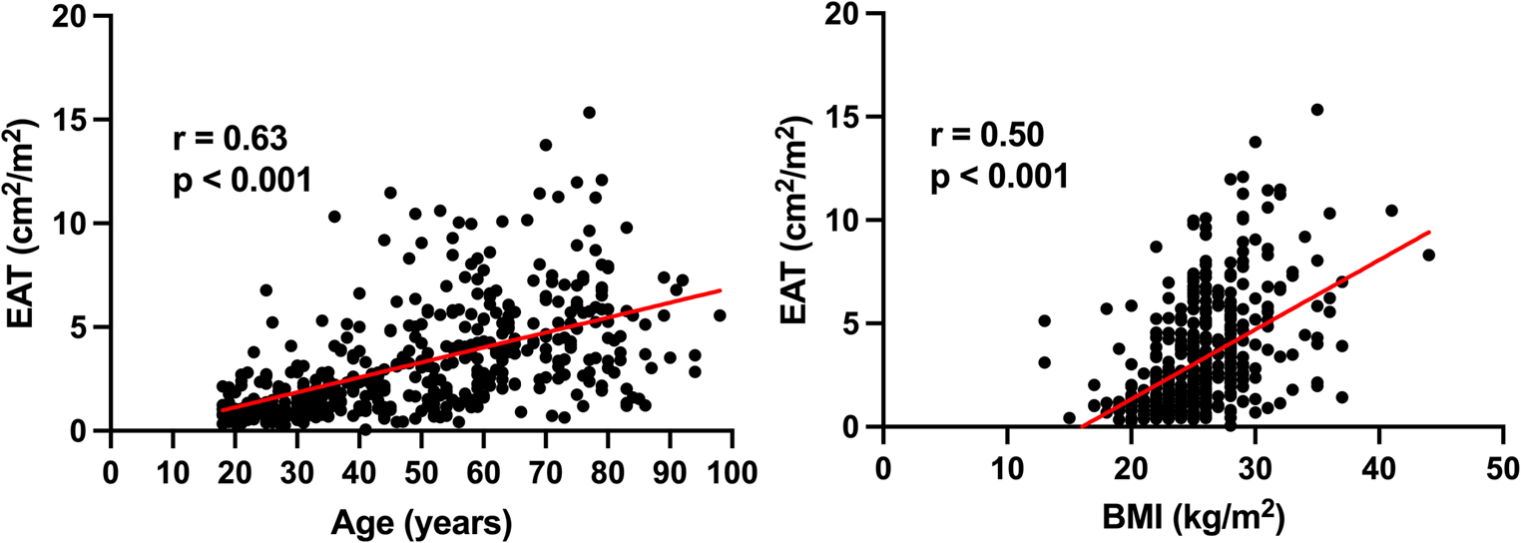
Spearman’s correlation coefficient between EAT index and age (**a**) and EAT index and BMI (**b**). The correlation coefficient was *r* = 0.63, *p* < 0.001 with age and *r* = 0.50, *p* < 0.001 with BMI, respectively

Median (IQR) EAT was 7.7 (3.5–14.2) cm^2^, whereas median EAT index was 2.4 (2.4–4.5) cm^2^/m^2^. EAT index correlated with age *r* = 0.63, *p* < 0.001 and BMI *r* = 0.50, *p* < 0.001 (Fig. 3).

### Associations with 24-hour mortality

In the Cox proportional hazard model, statistically significant univariable associations with 24-hour mortality were age, EAT index, and ISS, while sex and BMI were not significantly associated (Table 2a). Adjusted for multiple variables, 24-hour mortality was significantly associated with age and ISS, while EAT index revealed no significant association.

**Table 2:** Cox proportional hazard models of associations with 24-hour and 30-day mortality 24-hour mortality

Parameter	Univariable HR	95% CI	*p* value	Multivariable HR	95% CI	*p* value
**a) 24-hour mortality**						
Sex (1 = male)	1.21	0.58–2.52	0.607			
Age (years)	1.03	1.01–1.04	**0.004**	1.03	1.01–1.06	**0.003**
BMI (kg/m²)	0.98	0.9–1.08	0.707			
EAT (cm^2^/m^2^)	1.15	1.04–1.26	**0.007**	1.03	0.91–1.16	0.622
ISS (points)	1.07	1.05–1.09	**< 0.001**	1.08	1.06–1.1	**< 0.001**
**b) 30-day mortality**						
Sex (1 = male)	1.07	0.69–1.66	0.758			
Age (years)	1.02	1.01–1.03	**< 0.001**	1.03	1.02–1.04	**< 0.001**
BMI (kg/m²)	1.05	1–1.1	**0.036**	1.01	0.95–1.07	0.734
EAT (cm^2^/m^2^)	1.09	1.01–1.16	**0.013**	1.01	0.92–1.1	0.903
ISS (points)	1.07	1.05–1.08	**< 0.001**	1.07	1.06–1.08	**< 0.001**

HR, hazard ratio; CI, confidence interval; BMI, body mass index; EAT, epicardial adipose tissue; ISS, injury severity score; bold numbers indicate statistical significance (p   <  0.05).

### Associations with 30-day mortality

Univariable analysis of 30-day mortality revealed statistically significant associations with age, BMI, EAT index, and ISS, while sex was not significantly associated (Table 2b). Multivariable analysis confirmed age and ISS, as statistically significant associations, while BMI and EAT index were not significantly associated.

### Associations with ICU LOS in survivors

In univariable linear regression analysis, age, EAT index, and ISS were significantly associated with ICU LOS, while no significant associations were observed for sex and BMI (Table 3a). Multivariable analysis confirmed age and ISS as independent associations of ICU LOS, while EAT index was not significantly associated (Table 3b).

**Table 3:** Linear regression analysis of associations with the logarithms of ICU LOS and mechanical ventilation duration in survivors ICU LOS – univariable associations

Parameter	B	Beta	SE	t	95% CI for B	*p* value
**a) ICU LOS – unvariable associations**						
Sex (1 = male)	-0.034	-0.015	0.145	-0.234	-0.32 to 0.252	0.815
Age (years)	0.014	0.267	0.003	4.349	0.008 to 0.021	**< 0.001**
BMI (kg/m²)	0.025	0.093	0.017	1.479	-0.008 to 0.059	0.14
EAT (cm^2^/m^2^)	0.054	0.136	0.025	2.168	0.005 to 0.103	**0.031**
ISS (points)	0.058	0.726	0.004	14.607	0.05 to 0.065	**< 0.001**
**b) ICU LOS – multivariable associations**						
(Constant)	-0.217		0.167	-1.302	-0.545 to 0.111	0.194
Age (years)	0.018	0.343	0.003	6.086	0.012 to 0.024	**< 0.001**
EAT (cm^2^/m^2^)	0.005	0.014	0.023	0.240	-0.039 to 0.05	0.810
ISS (points)	0.061	0.765	0.004	16.441	0.053 to 0.068	**< 0.001**
**c) Mechanical ventilation duration – univariable associations**						
Sex (1 = male)	0.27	0.117	0.223	1.207	-0.17 to 0.709	0.228
Age (years)	0.021	0.4	0.005	4.233	0.011 to 0.031	**< 0.001**
BMI (kg/m²)	0.046	0.167	0.026	1.734	-0.006 to 0.097	0.084
EAT (cm^2^/m^2^)	0.095	0.237	0.038	2.461	0.019 to 0.17	**0.014**
ISS (points)	0.083	1.044	0.006	13.162	0.071 to 0.095	**< 0.001**
**d) Mechanical ventilation duration – multivariable associations**						
(Constant)	-2.526		0.269	-9.397	-3.054 to -1.997	**< 0.001**
Age (years)	0.026	0.483	0.005	5.313	0.016 to 0.035	**< 0.001**
EAT (cm^2^/m^2^)	0.027	0.067	0.036	0.739	-0.045 to 0.098	0.460
ISS (points)	0.088	1.104	0.006	14.711	0.076 to 0.099	**< 0.001**

ICU LOS, intensive care unit length of stay; B, unstandardized coefficient; Beta, standardized coefficient; SE, standard error; CI, confidence interval; BMI, body mass index; EAT, epicardial adipose tissue; ISS, injury severity score; all estimated parameters refer to the outcome on the log-scale; bold numbers indicate statistical significance (*p* < 0.05).

### Associations with mechanical ventilation duration in survivors

In univariable linear regression analysis, age, BMI, EAT index, and ISS were significantly associated with the duration of mechanical ventilation, while sex was not significantly associated (Table 3c). Multivariable analysis confirmed age and ISS as independent associations of mechanical ventilation duration in survivors, while BMI and EAT index were not significantly associated (Table 3d).

## Discussion

The present analysis sought to investigate the initial trauma CT of severely injured patients for novel by-product imaging markers. EAT as an important endocrine fat tissue parameter was elucidated for prognostic power in acute trauma patients requiring emergency tracheal intubation and mechanical ventilation. Univariable analysis revealed associations with 24-hour mortality, 30-day-mortality, and mechanical ventilation duration, but not with ICU LOS. After adjustment for multiple predictors including age, BMI, and ISS, no statistically significant prognostic effect was observed for EAT.

Several different studies have demonstrated the prognostic relevance of EAT throughout cardiovascular medicine, especially for coronary heart disease [7, 8, 11, 12]. The function of EAT in heart physiology include its role in cardiac metabolism with mechanical protection of coronary arteries, innervation, and potentially the cryoprotection [7, 8, 12]. In a recent study regarding diabetes patients, EAT volume was positively associated with age, BMI, pack-year history of smoking, and triglyceridemia but negatively correlated with HDL cholesterol level [13]. There was an important association between features of the metabolic syndrome and EAT expression which might also lead to worse outcomes in severely injured trauma patients with a reported odds ratio of 1.75 (95% CI 1.39–2.19) for in-hospital mortality in a recent large meta-analysis investigating the prognostic relevance of obesity and metabolic syndrome [14, 15]. Yet, there were was no systematic analysis regarding this special patient cohort to date.

Numerous studies have investigated potential imaging findings as prognostic markers in trauma patients derived from initial trauma CT [3–6]. For example, body composition analyses were investigated in different trauma populations. In a German study, 297 patients with multiple trauma and an ISS ≥ 16 points were investigated retrospectively [5]. The authors could show that the skeletal muscle index as a sarcopenia surrogate was associated with mortality with a reported hazard ratio of 0.98 (95% CI 0.97–0.99), which remained statistically significant in multivariable analysis. Another important imaging finding was coronary artery calcification, which and left main coronary artery calcification was independently associated with the development of any complication (odds ratio 3.9, 95% CI 1.7–8.9) in polytrauma patients in a cohort from the Netherlands [6]. The important role of the cardiovascular system on the prognosis in polytrauma patients is highlighted by these CT-defined findings.

Albeit there was a statistical signal for the prognostic relevance of EAT in the univariable analysis, it did not reach statistical significance in the multivariable analysis. This could be explained by several aspects. At first, the patient sample was not large enough to demonstrate a significant result. However, one could discuss that the effect size for EAT might also be considered as low and not clinically relevant in this scenario. Secondly, the effect by the EAT on mortality might be confounded by other prognostic features, such as age, ISS, and BMI status. Yet, one key finding of the present study is that the prognostic relevance of EAT in trauma patients can be considered as low.

The measurement of EAT is still not standardized in the literature. Some authors report only a thickness diameter derived from echocardiography or CT images [16]. The gold standard was highlighted as a whole volumetry to quantify all fat tissue surrounding the heart, which however, takes the most time to perform and, which can still be challenging to translate into clinical routine [9]. The area measurement employed in the present study was highly correlated with the volume of the EAT and can be considered an appropriate surrogate parameter with a reported correlation coefficient (*r* = 0.89, *p* < 0.0001), which is also faster to perform [9]. Beyond these measurement aspects, there is definite need for EAT threshold values and gender-specific cut-offs, as proposed for skeletal muscle index and visceral adipose tissue measurements in the field of CT body composition analysis [17, 18].

The merit of the present study is that the relevance of the EAT area on short-term outcomes is investigated in a systematic manner. The implications of the EAT area as surrogate imaging marker of the cardiovascular system on short-term outcomes in severely injured trauma patients remain nevertheless low.

The limitations of the present analysis are, first, its single-center retrospective design with possible known inherent bias, although the imaging analysis was performed blinded to the clinical information. Second, only patients requiring tracheal intubation, mechanical ventilation and who underwent whole-body CT were included in this analysis. The need for advanced airway management and mechanical ventilation during trauma resuscitation is a simple and pragmatic marker of considerable injury severity. Patients without tracheal intubation and those who did not receive initial whole-body CT were not investigated in the present analysis, which might be a selection bias. However, these exclusions led to a homogenous cohort with high overall injury severity and mortality rates. This has to be considered when interpreting the present results. Third, although the EAT measurement is a semiquantitative imaging analysis, investigator-related bias cannot be excluded completely. Fourth, traumatic injuries could lead to a false measurement of the EAT. For example, large pericardial effusions, pneumothorax, or intrathoracic herniation could reduce the space of the pericardial fat area and have an influence of the EAT measurement. However, in the present cohort, no relevant findings were identified, which could result in a relevant bias.

In conclusion, EAT index defined by initial polytrauma CT imaging showed univariable associations with short-term outcomes 24-hour mortality, 30-day mortality, ICU LOS and mechanical ventilation duration in severely injured trauma patients, but did not reach statistical significance in multivariable analysis, suggesting that the prognostic capability of this cardiac parameter is limited in this study cohort. Further validation studies are needed to confirm these findings.

## Data Availability

The dataset supporting the conclusions of this article is included within the article (Table S2).
